# Evaluation of Air Leak-related Complications in Segmentectomy: A Comparative Study with Lobectomy Using Goddard Score

**DOI:** 10.1093/icvts/ivag167

**Published:** 2026-06-03

**Authors:** Atsushi Kagimoto, Masaya Otabe, Takeshi Mimura

**Affiliations:** Department of General Thoracic Surgery, National Hospital Organization, Kure Medical Center and Chugoku Cancer Center, Kure, Hiroshima 737-0023, Japan; Department of General Thoracic Surgery, National Hospital Organization, Kure Medical Center and Chugoku Cancer Center, Kure, Hiroshima 737-0023, Japan; Department of General Thoracic Surgery, National Hospital Organization, Kure Medical Center and Chugoku Cancer Center, Kure, Hiroshima 737-0023, Japan

**Keywords:** segmentectomy, air leak, Goddard score, emphysema, non-small cell lung cancer

## Abstract

**Objectives:**

The relative incidence of postoperative air leak between lobectomy and segmentectomy remains unclear. This study aimed to examine how the presence and severity of emphysema—an established risk factor for air leak—affect the incidence of this complication in each surgical procedure.

**Methods:**

This retrospective study included patients who underwent lobectomy or segmentectomy for non-small cell lung cancer between April 2009 and March 2024. Patients were stratified into 2 groups based on the Goddard score (GS), a visual evaluation method for quantifying radiologic findings of emphysema on computed tomography (CT)with a maximum of 24 points: GS 0-5 and GS ≥6. The incidences of postoperative air leak-related complications (prolonged air leak, pleurodesis, reinsertion of the chest tube, or reoperation for air leak) were compared between the lobectomy and segmentectomy groups.

**Results:**

Overall, 747 patients were included in the study. Among 564 patients with GS 0-5, there were no significant differences in the incidence of postoperative air leak-related complications between lobectomy (5.7%) and segmentectomy (8.2%) groups (*P *= .275). Conversely, in 183 patients with GS ≥6, the incidence of such complications was significantly lower in the segmentectomy group (7.0%) compared to the lobectomy group (18.3%) (*P *= .036).

**Conclusions:**

In patients with moderate to severe emphysema (GS ≥6), segmentectomy was associated with a lower incidence of postoperative air leak-related complications than lobectomy. This may reflect the advantage of greater parenchymal preservation; however, these findings should be interpreted cautiously given the observational study design.

## Graphical abstract

**Figure:**
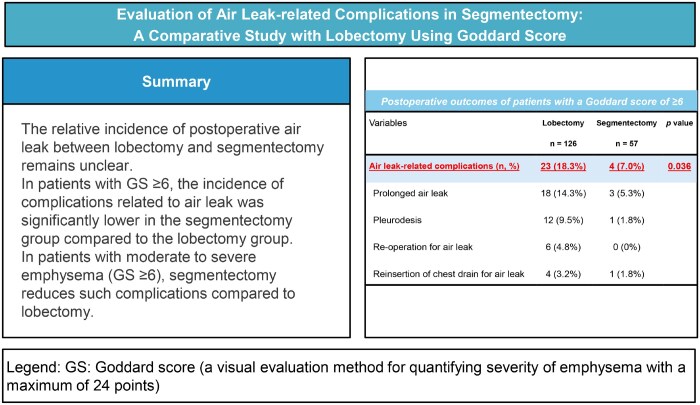

## INTRODUCTION

Radiologic findings of emphysema on computed tomography (CT) have been identified as a risk factor for postoperative air leak.^1^ We previously demonstrated that the Goddard score (GS), a visual evaluation method for radiologic findings of emphysema on CT (maximum of 24 points) that can be easily calculated without specialized software,^2^ is useful for predicting postoperative air leak-related complications after lobectomy.^3^ In that study, radiologic findings of emphysema with a GS of ≥6 were identified as a significant predictor of postoperative air leak-related complications. However, the study included only patients who underwent lobectomy and did not evaluate whether the incidence of air leak-related complications varied depending on the surgical procedure.

Recently, a prospective study demonstrated that segmentectomy is superior to lobectomy in terms of overall survival for patients with peripherally located small non-small cell lung cancer (NSCLC),^4^ and segmentectomy has become one of the standard treatments. The advantages of preserving the lung parenchyma have resulted in favourable pulmonary function^5^ and improved prognosis. However, studies have reported inconsistent findings regarding the frequency of air leak-related complications between segmentectomy and lobectomy. In the secondary analysis of a prospective study, segmentectomy was associated with a higher incidence of prolonged air leak and chest drain reinsertion due to air leak,^6^ whereas a study using the Japanese national database showed a lower incidence of prolonged air leak after segmentectomy compared to lobectomy.^7^ Furthermore, these studies did not account for the presence of emphysema, which is a risk factor for air leaks, and no study has directly compared the incidence of air leaks between segmentectomy and lobectomy in patients with emphysema.

Therefore, this study aimed to evaluate whether the influence of surgical procedure on air leak-related complications varies according to the severity of emphysematous change, as assessed by GS, instead of focusing solely on a direct comparison between lobectomy and segmentectomy.

## METHODS

### Patients

This retrospective study was approved by the Institutional Review Board of the Kure Medical Center and Chugoku Cancer Center (registration number: No.2024-94), and the requirement for informed consent was waived. The storage of participant data for multiple, indefinite research uses met the standards set forth in the WMA Declaration of Taipei. This study included patients who underwent lobectomy or segmentectomy for NSCLC between April 2009 and March 2024 at the National Hospital Organization, Kure Medical Center and Chugoku Cancer Center. Patients who had undergone preoperative neoadjuvant therapy, open thoracotomy, or simultaneous lobectomy and segmentectomy were excluded.

### Preoperative examinations

Chest CT, whole-body positron emission tomography with [18F]-fluoro-2-deoxy-D-glucose, brain magnetic resonance imaging, and pulmonary function test were performed to determine the clinical stage (determined based on the 8th edition of the TNM classification)^8^ and treatment strategies.

### Evaluation of Goddard score

Goddard score is a visual assessment method for quantifying radiologic emphysematous changes on CT, in which each lung is divided into upper, middle, and lower zones, totalling 6 zones. It is scored based on the extent of low-attenuation areas in each zone. Scores ranged from 0 (no emphysema) to 4 (≥75% involvement) per zone, with a maximum total score of 24.^2^ The details of this method are described in our previous study.^3^ The GS was evaluated during preoperative conferences through consensus among thoracic surgeons, and the results were prospectively recorded in our institutional database. Based on this scoring system, patients were categorized into 2 groups: those with a GS of 0-5 and those with a GS of ≥6. The cutoff value was determined according to the results of our previous study.^3^

### Indications for lobectomy and segmentectomy

Intentional segmentectomy is performed for patients with lesions located in the outer third of the lung field and measuring ≤20 mm in diameter. Segmentectomy is also performed with passive intent in patients who are intolerant of lobectomy because of comorbidities, poor pulmonary function, or low performance status. The attending surgeon made the final decision regarding the procedure.

### Surgical procedure

Briefly, surgical procedures were performed by complete video-assisted thoracoscopic surgery (cVATS), as described in our previous study.^3^^,^^9^ Hybrid VATS was also used in the early phases of the study. In cases of segmentectomy, the intersegmental plane was identified using the inflation-deflation technique with jet ventilation or intravenous indocyanine green, and then divided using surgical staplers and a microwave surgical instrument.^10^

After lung resection, a water-sealing test was performed using distilled water at a pressure of 20 cmH_2_O. If an air leak was detected, the affected site was managed by soft coagulation or reinforcement with fibrin glue and a polyglycolic acid sheet. A chest tube (either a 19 Fr. silicon drain or a 20 Fr. polyvinyl chloride chest tube) was inserted before the chest was closed. The chest tube was managed with a suction pressure of -7 cm H_2_O postoperatively.

### Statistical analysis

As all variables were non-normally distributed, they are expressed as median (interquartile range [IQR]) and analysed using the Wilcoxon rank-sum test. Categorical variables were compared using the chi-squared test. Numbers (n) and percentages were calculated for categorical variables. In this study, air leak-related complications, defined as prolonged air leak for ≥5 days, pleurodesis, reinsertion of chest drain, or reoperation for air leak, were defined as events. The incidence of air leak-related complications following lobectomy and segmentectomy was compared between patients with a GS of 0-5 and those with a GS of ≥6.

An interaction analysis was performed using multivariable logistic regression models. The model included surgical procedure, GS (as a continuous variable), and their interaction term (surgical procedure × GS) as independent variables, with the occurrence of air leak-related complications as the dependent variable.

Multivariable logistic regression analyses were conducted using variables that were significant in univariable analysis to identify risk factors for air leak-related complications in patients who underwent segmentectomy and lobectomy, respectively. Because GS was a predictor of air leak-related complications in patients who underwent lobectomy but not in those undergoing segmentectomy, a receiver operating characteristic (ROC) curve analysis was performed specifically in the lobectomy subgroup to assess the predictive value of GS as an exploratory analysis.

JMP version 14 (SAS Institute, Cary, NC, USA) was used for all statistical analyses; *P *< .05 was considered statistically significant.

## RESULTS

**Figure 1.**
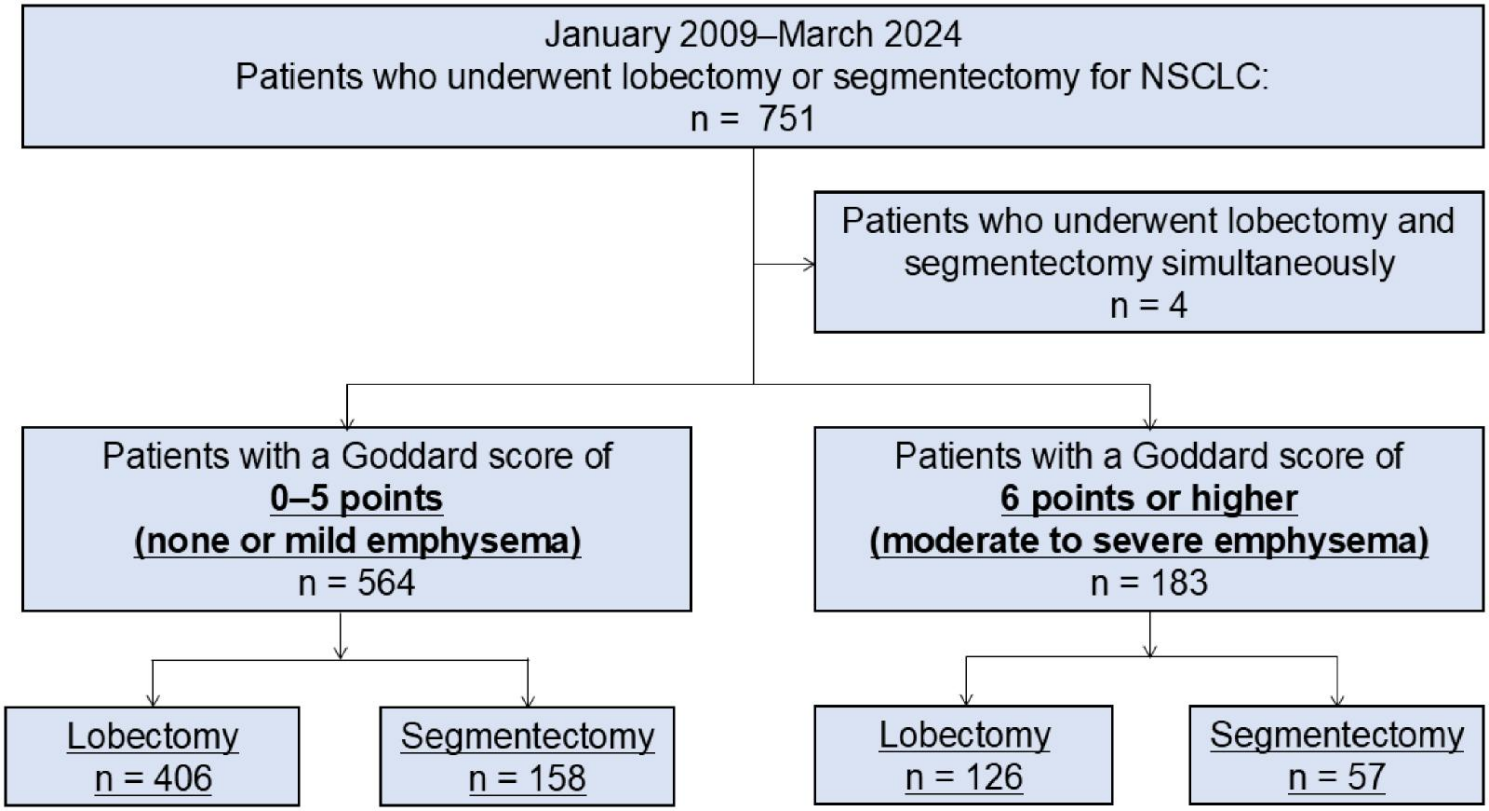
Flowchart of Patient Selection. Overall, 747 patients were included in this study. Among them, 564 patients had a Goddard score (GS) of 0-5 points, and 183 patients had a GS of ≥6 points. In the GS 0-5 group, 406 patients underwent lobectomy, and 158 underwent segmentectomy. In the GS ≥6 group, lobectomy and segmentectomy were performed in 126 and 57 patients, respectively. Abbreviation: NSCLC, non-small cell lung cancer.

This study included 747 patients. Among them, 564 patients (75.5%) had a GS of 0-5 points, and 183 patients (24.5%) had a GS of ≥6 points. In the GS 0-5 group, 406 patients (72.0%) underwent lobectomy, and 158 (28.0%) underwent segmentectomy. In the GS ≥6 group, lobectomy and segmentectomy were performed in 126 (68.9%) and 57 patients (31.1%), respectively (**Figure 1**).

### Interaction analysis, multivariable analysis, and ROC curve

The interaction analysis between surgical procedure and GS did not demonstrate a statistically significant interaction (*P *= .202), indicating that GS did not significantly modify the effect of lobectomy versus segmentectomy on air leak-related complications (**Table S1**). In addition to the primary interaction analysis, we conducted stratified univariable and multivariable analyses to explore factors associated with air leak-related complications. These analyses were performed solely for exploratory and descriptive purposes and should not be interpreted as evidence of effect modification. Among patients who underwent lobectomy, GS and smoking burden were associated with air leak-related complications in the multivariable model (**Table S2**). These findings are descriptive and should be interpreted cautiously. Among patients who underwent segmentectomy, no variable other than CCI reached statistical significance in the univariable analysis (**Table S3**). The distribution of surgical procedures according to surgical era and GS subgroup is shown in **Table S4** and **Figure S1**. ROC curve was generated for patients who underwent lobectomy and revealed that the GS is a significant predictor (area under the curve, 0.677; 95% confidence interval [CI], 0.596-0.758; *P *< .001, **Figure S2**). Based on the Youden index, the optimal cutoff value of GS in the present study was GS of 4 (specificity, 71.6%; sensitivity, 60.9%). However, we used a GS of 6 (specificity, 78.8%; sensitivity, 50.0%) as the cutoff point of the present study, considering consistency with our previous studies, specificity, and clinical significance (a threshold that is too low increases the proportion of high-risk cases).

### Outcomes in patients with low emphysema burden (GS 0-5)

The characteristics of the patients with a GS of 0-5 are shown in **Table 1**. In patients with no or mild emphysematous changes, the incidence of postoperative air leak-related complications was 5.7% in the lobectomy group and 8.2% in the segmentectomy group, with no statistically significant difference (*P *= .275, **Table 2**). Similarly, there were no significant differences in the operative time (*P *= .191) and length of hospital stay (*P *= .266) between the 2 groups. The drainage duration was shorter in segmentectomy (*P *= .028). Among patients who underwent lobectomy, prolonged air leak occurred in 18 patients (4.4%), pleurodesis was performed in 12 (3.0%), and chest drain reinsertion was required in 6 (1.5%). In the segmentectomy group, prolonged air leak was observed in 11 patients (7.0%), pleurodesis in 9 (5.7%), and chest drain reinsertion in 2 (1.3%). Collectively, in patients with limited or no emphysema, postoperative outcomes were largely comparable between lobectomy and segmentectomy.

**Table 1.** Patient Characteristics of Patients with a Goddard Score of 0-5

Variables	Lobectomy *n* = 406 (72.0%)	Segmentectomy *n* = 158 (28.0%)	*P* value
Age, (years) (median, IQR)	71 (65-75.75)	72.5 (67-77)	.009
Sex (*n*, %)			.875
Male	206 (50.7%)	79 (50.0%)	
Female	200 (49.3%)	79 (50.0%)	
Smoking history (*n*, %)	221 (54.4%)	85 (53.8%)	.892
BI (median, IQR)	155 (0-740)	60 (0-600)	.381
CCI (median, IQR)	1 (0-2)	1 (0-2)	.015
Pulmonary function			
%VC (%) (median, IQR)	103.8 (92.12-114.6)	96.5 (85.45-109.05)	.001
FEV_1_% (%) (median, IQR)	79.36 (74.50-82.93)	78.01 (73.27-81.93)	.111
%DLCO (%) (median, IQR)	93.1 (75.47-110.52) (*n* = 242)	95.0 (80.35-111.68) (*n* = 120)	.501
Radiological findings of IP (*n*, %)	62 (15.3%)	13 (8.3%)	.021
Radiological findings of emphysema (*n*, %)	104 (25.6%)	41 (26.0%)	.935
Goddard score (median, IQR)	0 (0-1)	0 (0-1)	.960
Approach (*n*, %)			.077
hybrid VATS	85 (20.9%)	23 (14.6%)	
complete VATS	321 (79.1%)	135 (85.4%)	
Tumour location (*n*, %)			< .001
Right upper lobe	142 (35.0%)	44 (27.8%)	
Right middle lobe	37 (9.1%)	0 (0%)	
Right lower lobe	98 (24.1%)	23 (14.6%)	
Left upper lobe	83 (20.4%)	64 (40.5%)	
Left lower lobe	46 (11.3%)	27 (17.1%)	
Clinical stage (*n*, %)			< .001
0	8 (2.0%)	16 (10.1%)	
IA	263 (64.8%)	138 (87.3%)	
IB	57 (14.0%)	8 (5.1)	
II	48 (11.8%)	4 (2.5%)	
III	30 (7.4%)	0 (0%)	

Abbreviations: BI, Brinkman index; CCI, Charlson comorbidity index; DLCO, diffusion capacity of the lungs for carbon monoxide; FEV_1_, forced expiratory volume in 1 second/forced vital capacity; IP, interstitial pneumonia; IQR, interquartile range; VATS, video-assisted thoracic surgery; VC, vital capacity of % predicted.

**Table 2.** Operative and Postoperative Outcomes of Patients with a Goddard Score of 0-5

Variables	Lobectomy, *n* = 406 (72.0%)	Segmentectomy, *n* = 158 (28.0%)	*P* value
Operation time (median, IQR) (min)	206 (171-241.75)	212.5 (175.25-243.75)	.191
Blood loss (median, IQR) (mL)	30 (5-87.5)	10 (5-50)	.035
Drainage duration (median, IQR) (days)	2 (2-3)	2 (2-2.75)	.028
Length of hospitalization (median, IQR) (days)	7 (6-9)	8 (6-10)	.266
Air leak-related complications (*n*, %)	23 (5.7%)	13 (8.2%)	.275
Prolonged air leak	18 (4.4%)	11 (7.0%)	.287
Pleurodesis	12 (3.0%)	9 (5.7%)	.139
Re-operation for air leak	1 (0.3%)	1 (0.6%)	.482
Reinsertion of chest drain for air leak	6 (1.5%)	2 (1.3%)	1.000

Abbreviation: IQR, interquartile range.

### Outcomes in patients with moderate to severe emphysema (GS ≥6)

**Table 3** presents the characteristics of patients with a GS of ≥6. Among patients with more pronounced emphysematous changes, the incidence of postoperative air leak-related complications was significantly lower in the segmentectomy group (7.0%) than in the lobectomy group (18.3%, *P *= .036, **Table 4**). There were no significant differences in drainage duration (*P *= .176) or length of hospital stay (*P *= .662) between the groups. The amount of intraoperative blood loss was significantly lower in the segmentectomy group compared with the lobectomy group (*P *= .014). In the lobectomy group, prolonged air leak occurred in 18 patients (14.3%), pleurodesis was required in 12 (9.5%), chest drain reinsertion in 4 (3.2%), and reoperation in 6 (4.8%). Contrastingly, among patients who underwent segmentectomy, prolonged air leak occurred in 3 patients (5.3%), pleurodesis and drain reinsertion were each required in 1 (1.8%), and no reoperations were performed. These results indicate that, in this cohort, the incidence of air leak-related complications was lower after segmentectomy than after lobectomy among patients with moderate-to-severe emphysema. The association of the differences in the incidence of air leak-related complications between lobectomy and segmentectomy and GS is shown in **Figure S3**. The incidence of air leak-related complications was higher for those who underwent lobectomy than segmentectomy in all groups except in patients with a GS of 0-5.

**Table 3.** Patient Characteristics of Patients with a Goddard Score of ≥6

Variables	Lobectomy, *n* = 126 (68.9%)	Segmentectomy, *n* = 57 (31.1%)	*P* value
Age, (years) (median, IQR)	73 (67-76.75)	74 (70-77)	.104
Sex (*n*, %)			.257
Male	122 (96.8%)	53 (93.0%)	
Female	4 (3.1%)	4 (7.0%)	
Smoking history (*n*, %)	126 (100%)	57 (100%)	NA
BI (median, IQR)	1030 (800-1200)	1100 (855-1920)	.103
CCI (median, IQR)	1 (0.25-2)	2 (1-3)	.012
Pulmonary function			
%VC (%) (median, IQR)	99.7 (85.5-110.9)	92.4 (78.7-102.2)	.015
FEV_1_% (%) (median, IQR)	72.3 (65.64-78.48)	68.92 (59.35-79.25)	.117
%DLCO (%) (median, IQR)	86.8 (66.6-104.2) (*n* = 93)	76.55 (59.98-97.2) (*n* = 50)	.340
Radiological findings of IP (*n*, %)	39 (31.0%)	16 (28.1%)	.693
Radiological findings of emphysema (*n*, %)	128 (100%)	57 (100%)	NA
Goddard score (median, IQR)	8 (6-12)	8 (6-12)	.831
Approach (*n*, %)			.281
hybrid VATS	31 (24.6%)	10 (17.5%)	
complete VATS	95 (75.4%)	47 (82.5%)	
Tumour location (*n*, %)			.002
Right upper lobe	52 (41.3%)	13 (22.8%)	
Right middle lobe	7 (5.6%)	0 (0%)	
Right lower lobe	28 (22.2%)	12 (21.1%)	
Left upper lobe	22 (17.5%)	24 (42.1%)	
Left lower lobe	17 (13.5%)	8 (14.0%)	
Clinical stage (*n*, %)			.001
0	1 (0.8%)	2 (3.5%)	
IA	61 (48.4%)	40 (70.2%)	
IB	26 (20.6%)	10 (17.5%)	
II	25 (19.8%)	5 (8.8%)	
III	13 (10.3%)	0 (0%)	

Abbreviations: BI, Brinkman index; CCI, Charlson comorbidity index; DLCO, diffusion capacity of the lungs for carbon monoxide; FEV_1_, forced expiratory volume in 1 second/forced vital capacity; IP, interstitial pneumonia; IQR, interquartile range; VATS, video-assisted thoracic surgery; %VC, vital capacity of % predicted.

**Table 4.** Operative and Postoperative Outcomes of Patients with a Goddard Score of ≥6

Variables	Lobectomy, *n* = 126 (68.9%)	Segmentectomy, *n* = 57 (31.1%)	*P* value
Operation time (median, IQR) (min)	214 (179.75-253.75)	221 (179-246)	.832
Blood loss (median, IQR) (mL)	50 (5-118.75)	20 (5-65)	.014
Drainage duration (median, IQR) (days)	2 (2-5)	2 (2-3)	.176
Length of hospitalization (median, IQR) (days)	9 (7-13)	8 (7-12)	.662
Air leak-related complications (*n*, %)	23 (18.3%)	4 (7.0%)	.036
Prolonged air leak	18 (14.3%)	3 (5.3%)	.085
Pleurodesis	12 (9.5%)	1 (1.8%)	.067
Re-operation for air leak	6 (4.8%)	0 (0%)	.179
Reinsertion of chest drain for air leak	4 (3.2%)	1 (1.8%)	1.000

Abbreviation: IQR, interquartile range.

## DISCUSSION

In the present study, among patients with a GS of 0-5, the incidence of air leak-related complications did not differ significantly between those who underwent lobectomy and those who underwent segmentectomy. However, in patients with a GS of ≥6, indicating moderate to severe emphysema, the incidence of air leak-related complications was significantly lower in the segmentectomy group than in the lobectomy group. These results indicate an association between segmentectomy and a lower incidence of postoperative air leak-related complications in patients with emphysematous lungs.

Although segmentectomy involves dividing the intersegmental lung parenchyma and requires more peripheral dissection of the pulmonary vessels and bronchi, which could theoretically increase the risk of air leaks, our results do not support this assumption. In fact, the secondary analysis of a phase III trial by Suzuki et al^6^ reported higher rates of prolonged air leak and chest tube reinsertion following segmentectomy. However, this study did not explicitly assess the presence or severity of emphysematous changes, which are well-established risk factors for air leak. Therefore, the potential benefits of segmentectomy in patients with emphysema have not been fully understood. One possible explanation for our findings is the difference in residual lung volume between the procedures. Lobectomy can create a large dead space due to extensive removal of the lung parenchyma, which may lead to persistent air leaks caused by enhanced negative intrathoracic pressure. In contrast, segmentectomy preserves more lung tissue and results in smaller dead space, which may be especially advantageous in hyperinflated emphysematous lungs. The reduced traction on fragile lung tissue may contribute to better sealing not only of the staple line but also of the dissected areas near the pulmonary hilum, thereby lowering the risk of postoperative air leak.

Miura et al^11^ also demonstrated that sublobar resection is a predictive factor for a low incidence of postoperative respiratory complications in patients with a low-attenuation area (%LAA) >1.1% on CT. However, their study grouped wedge resection and segmentectomy together despite differences in invasiveness and air leak risk.^12^ Moreover, that study did not focus specifically on air leak-related complications, and the %LAA threshold may have included patients with only minimal emphysematous changes. In contrast, our study used GS, a simple and widely applicable visual scoring method, to stratify patients by emphysema severity and focused specifically on air leak-related complications. Thus, our findings offer novel insights into the surgical management of patients with emphysema.

In clinical practice, it is sometimes challenging to determine whether to prioritize oncological outcomes or the risk of postoperative complications, particularly in cases where segmentectomy is performed with passive intent. Although oncological radicality should remain the main determinant in surgical decision-making, the GS may serve as a supplementary parameter to help balance oncological and functional considerations, particularly regarding postoperative complications such as air leak. The present findings suggest that surgeons do not need to avoid segmentectomy solely because of concerns regarding air leak.

This study had some limitations. First, it was a retrospective study conducted at a single institution. The attending surgeon decided the surgical procedure based on the patient’s clinical condition, so a certain level of selection bias cannot be excluded. In particular, differences in clinical stage and patient characteristics between the lobectomy and segmentectomy groups suggest the presence of confounding by indication, which may have influenced postoperative outcomes. Although multivariable analyses were performed, these analyses were exploratory and may not have fully accounted for all relevant confounders. The number of patients with a high GS value was relatively small, which may have reduced the statistical power of subgroup analyses and increased the risk of type II error. In the present study, the incidence of air leak-related complications was relatively low in patients with high GS who underwent segmentectomy, and this finding might reflect insufficient statistical power due to the limited sample size. The second limitation is the evolution of operative techniques over the study period. In recent years, we introduced additional devices such as the microwave surgical instrument,^10^ which has been shown to reduce postoperative air leakage. Advances in thoracoscopic visualization and instruments may also have contributed to improvements in air leak-related outcomes. However, these technological refinements were applied to both segmentectomy and lobectomy and, therefore, are unlikely to have biased the relative comparison between procedures. Furthermore, the year of operation was not a predictor of air leak-related complications. Third, we acknowledge that the GS represents a continuous and semi-quantitative measure, and that any categorization inevitably involves some arbitrariness. Importantly, the key clinical implication of our study remains that, regardless of the specific GS value, preserving a greater amount of functional lung parenchyma, such as through segmentectomy, may reduce the risk of air leak-related complications in patients with emphysematous lungs. Furthermore, the GS was assessed bilaterally, and we could not analyse the degree of emphysema separately for the operated and contralateral sides. However, because emphysematous changes are usually bilateral and symmetrical, we believe that the influence of the contralateral side on the overall score was likely minimal. Fourth, the degree of fissure completeness, which is known to influence postoperative air leak, was not assessed owing to the unavailability of data for the present cohort. Despite these limitations, to our knowledge, this study is the first to evaluate the impact of emphysema severity, quantified by the GS, on the relationship between surgical procedure and postoperative air leak. Our findings suggest that the GS, a simple visual CT-based index, may help identify patients for whom segmentectomy is a more appropriate option than lobectomy, specifically in terms of minimizing the risk of air leak-related complications. Importantly, across all analytical approaches, segmentectomy tended to show a lower incidence of air leak-related complications than lobectomy, even in patients with greater emphysematous changes, supporting the clinical value of parenchyma-sparing resection.

## CONCLUSION

Although the incidence of postoperative air leak-related complications did not differ between lobectomy and segmentectomy in patients with a GS of 0-5, segmentectomy was associated with a lower incidence of such complications in patients with a GS of ≥6. This observation may reflect the advantage of greater parenchymal preservation with segmentectomy; however, given the lack of a significant interaction between surgical procedure and GS, as well as potential selection bias inherent to this retrospective study, these findings should be interpreted with caution and considered hypothesis-generating.

## Supplementary Material

ivag167_Supplementary_Data

## Data Availability

The data supporting this article will be shared upon reasonable request to the corresponding author.

## Abbreviations

CI: confidence interval
CT: computed tomography
GS: Goddard score
IQR: interquartile range
LAA: low attenuation area
NSCLC: non-small cell lung cancer
ROC: receiver operating characteristic
VATS: video-assisted thoracoscopic surgery
