# Supplementary material for: Evaluation of Air Leak-related Complications in Segmentectomy: A Comparative Study with Lobectomy Using Goddard Score
Source: Interdiscip Cardiovasc Thorac Surg. 2026 Jun 3;41(6):ivag167. doi: 10.1093/icvts/ivag167 (PMC13264385; doi:10.1093/icvts/ivag167)
Supplement: ivag167_Supplementary_Data [file ivag167_supplementary_data.zip › Supplementary_Data/Supplemental Figure legend.docx]

**Supplemental Figure legend**

**Supplementary Materials, Figure S1**

Stacked bar charts showing the annual distribution of lobectomy and segmentectomy in patients with a (A) Goddard score (GS) of 0–5 and (B) GS ≥6. Similar temporal trends were observed in both groups.

**Supplementary Materials, Figure S2**

Receiver operating characteristic curve of Goddard score (GS) predicting air leak-related complications in patients who underwent lobectomy. The GS is a significant predictor (area under the curve, 0.677 [95% confidence interval, 0.596–0.758]; *p* < 0.001). Based on the Youden index, the optimal cutoff value of GS in the present study was GS of 4 (Specificity, 71.6%; Sensitivity, 60.9%). However, we use GS of 6 (Specificity, 78.8%; Sensitivity, 50.0%) as cutoff point of the present study considering consistency with our previous studies, specificity, and clinical significance (a threshold that is too low increases the proportion of high-risk cases).

**Supplementary Materials, Figure S3**

The association of the differences in the incidence of air leak-related complications between lobectomy and segmentectomy and GS. Although there was no statistical significance, the incidences of air leak-related was higher in lobectomy in patients with a GS of 6-10 (lobectomy, 17.9%; segmentectomy, 5,6%, *p* = 0.092), 11-15 (lobectomy, 22.2%; segmentectomy, 9.1%; *p* = 0.344), 16- (lobectomy, 13.3%; segmentectomy, 10.0%; *p* = 0.738).
