# Supplementary material for: Evaluation of Air Leak-related Complications in Segmentectomy: A Comparative Study with Lobectomy Using Goddard Score
Source: Interdiscip Cardiovasc Thorac Surg. 2026 Jun 3;41(6):ivag167. doi: 10.1093/icvts/ivag167 (PMC13264385; doi:10.1093/icvts/ivag167)
Supplement: ivag167_Supplementary_Data [file ivag167_supplementary_data.zip › Supplementary_Data/Supplemental Tables.docx]

**Supplementary Materials, Table S1. Interaction analysis of Goddard score and surgical procedure for predicting air leak-related complications**

| variable | β coefficient | SE | Wald z | p value | Adjusted OR (95% CI) |
| --- | --- | --- | --- | --- | --- |
| Intercept | −2.760 | 0.205 | −13.45 | <0.001 | — |
| Goddard score (per 1-point increase) | 0.104 | 0.027 | 3.87 | <0.001 | 1.11 (1.05–1.17) |
| Procedure (Lobectomy = 0, Segmentectomy = 1) | 0.169 | 0.373 | 0.45 | 0.650 | 1.18 (0.57–2.46) |
| Interaction term (Goddard × Procedure) | −0.067 | 0.052 | −1.28 | 0.202 | 0.94 (0.84–1.04) |

CI, confidence interval; OR, odds ratio; SE, standard error

**Supplementary Materials, Table S2. Univariable and multivariable analysis to predict air leak-related complications in patients who underwent lobectomy**

|  | Univariable analysis | | Multivariable analysis | | | |
| --- | --- | --- | --- | --- | --- | --- |
| Variables | OR (95% CI) | *p* value | | OR (95% CI) | *p* value |  |
| Age (≥75 y/<75 y) | 2.260 (1.170–4.350) | 0.015 | | 1.890 (0.957–3.740) | 0.067 |  |
| BI (≥1000/<1000) | 1.760 (1.760–6.030) | < 0.001 | | 2.190 (1.050–4.570) | 0.037 |  |
| CCI (≥2/<2) | 1.660 (0.903–3.040) | 0.103 | |  |  |  |
| %VC (<80%/≥80%) | 0.7190 (0.214–2.410) | 0.593 | |  |  |  |
| FEV_1_% (<70%/≥70%) | 2.520 (1.310–4.820) | 0.005 | | 1.530 (0.691–3.370) | 0.296 |  |
| Years (2018-/-2017) | 0.603 (0.328–1.110) | 0.102 | |  |  |  |
| Location |  |  | |  |  |  |
| RM/RU | * | NA | |  |  |  |
| RL/RU | 0.748 (0.349–1.600) | 0.454 | |  |  |  |
| LU/RU | 0.823 (0.374–1.810) | 0.628 | |  |  |  |
| LL/RU | 0.391 (0.113-1.350) | 0.138 | |  |  |  |
| Radiological finding of IP (yes/no) | 0.690 (0.402–1.970) | 0.773 | |  |  |  |
| Goddard score (continuous value) | 1.110 (1.050–1.170) | < 0.001 | | 1.080 (1.010–1.160) | 0.023 |  |
| Clinical Stage (≥IB/≤IA) | 1.720 (0.935–3.150) | 0.083 | |  |  |  |
| Approach (cVATS/hVATS) | 0.877 (0.431–1.790) | 0.717 | |  |  |  |

SD, standard deviation; BI, Brinkman index; CCI, Charlson Comorbidity Index; VC, vital capacity of percent predicted; FEV1, forced expiratory volume in 1 second/forced vital capacity; RM, right middle lobe; RU, right upper lobe; LU, left upper lobe; LL, left lower lobe; IP, interstitial pneumonia; cVATS, complete video-assisted thoracic surgery; hVATS, hybrid video-assisted thoracic surgery; OR, odds ratio; CI, confidence interval

**Supplementary Materials, Table S3. Univariable analysis to predict air leak-related complications in patients who underwent segmentectomy**

|  | Univariable analysis | |  |
| --- | --- | --- | --- |
| Variables | OR (95% CI) | *p* value | |
| Age (≥75 y/<75 y) | 1.930 (0.562–6.620) | 0.296 | |
| BI (≥1000/<1000) | 1.750 (0.615–4.990) | 0.294 | |
| CCI (≥2/<2) | 5.380 (1.500–19.300) | 0.010 | |
| %VC (<80%/≥80%) | 0.932 (0.255–3.410) | 0.916 | |
| FEV_1_% (<70%/≥70%) | 1.660 (0.583–4.720) | 0.343 | |
| Years (2018-/-2017) | 0.872 (0.295–2.580) | 0.805 | |
| Location |  |  | |
| RM/RU |  |  | |
| RL/RU | 0.630 (0.116–3.440) | 0.594 | |
| LU/RU | 1.180 (0.376–3.730) | 0.772 | |
| LL/RU | 0.306 (0.034–2.730) | 0.289 | |
| Radiological finding of IP (yes/no) | 1.420 (0.381–5.270) | 0.603 | |
| Goddard score (continuous value) | 1.040 (0.951–1.130) | 0.402 | |
| Clinical Stage (≥IB/≤IA) | 0.379 (0.048–2.980) | 0.356 | |
| Approach (cVATS/hVATS) | 0.833 (0.226–3.080) | 0.784 | |

SD, standard deviation; BI, Brinkman index; CCI, Charlson Comorbidity Index; VC, vital capacity of percent predicted; FEV1, forced expiratory volume in 1 second/forced vital capacity; RM, right middle lobe; RU, right upper lobe; LU, left upper lobe; LL, left lower lobe; IP, interstitial pneumonia; cVATS, complete video-assisted thoracic surgery; hVATS, hybrid video-assisted thoracic surgery; OR, odds ratio; CI, confidence interval.

**Supplementary Materials, Table S4. Distribution of surgical procedures according to surgical era in each Goddard score group**

| Patients with a GS of 0–5 | | | Patients with a GS of ≥6 | | |
| --- | --- | --- | --- | --- | --- |
| Years | Lobectomy | Segmentectomy | | Lobectomy | Segmentectomy |
| -2017 (n, %) | 241 (82.0%) | 53 (18.0%) | | 51 (79.7%) | 13 (20.3%) |
| 2018- (n, %) | 165 (61.1%) | 105 (38.9%) | | 75 (63.0%) | 44 (37.0%) |

GS, Goddard score.
