# Supplementary figures and images for: Evaluation of Air Leak-related Complications in Segmentectomy: A Comparative Study with Lobectomy Using Goddard Score
Source: Interdiscip Cardiovasc Thorac Surg. 2026 Jun 3;41(6):ivag167. doi: 10.1093/icvts/ivag167 (PMC13264385; doi:10.1093/icvts/ivag167)

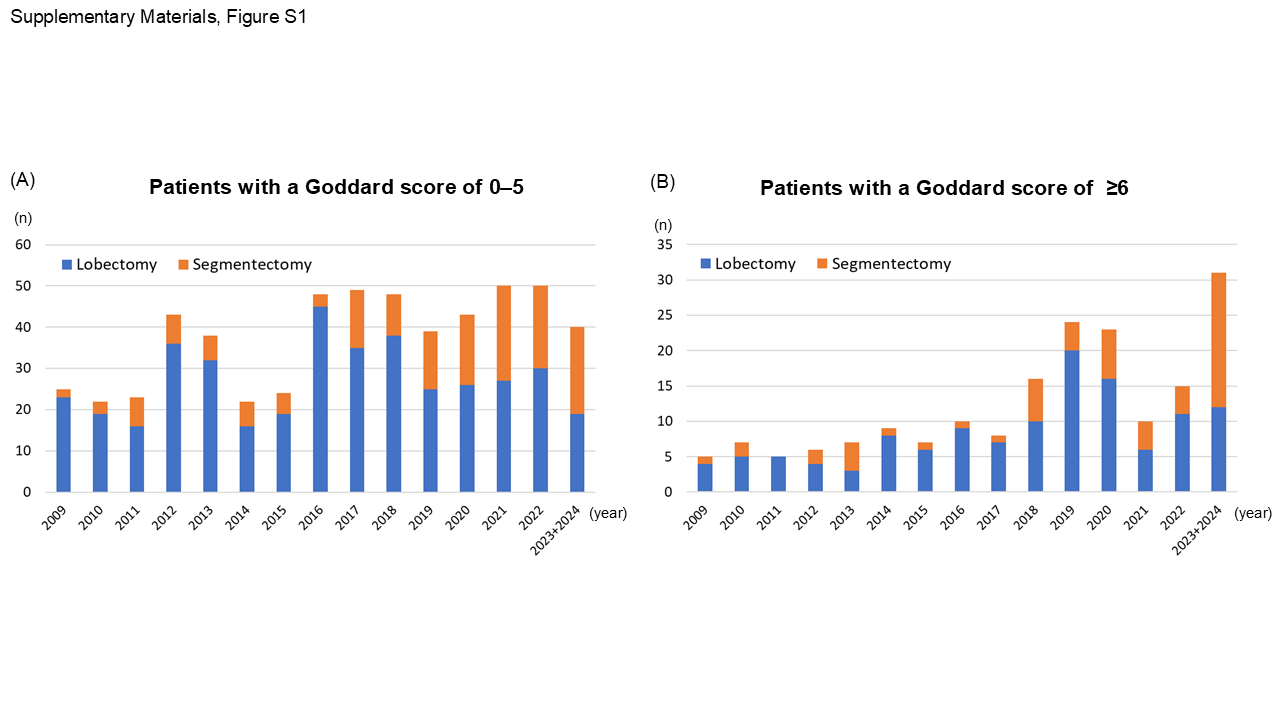

Supplement: ivag167_Supplementary_Data [file ivag167_supplementary_data.zip › Supplementary_Data/Figure S1.tif]

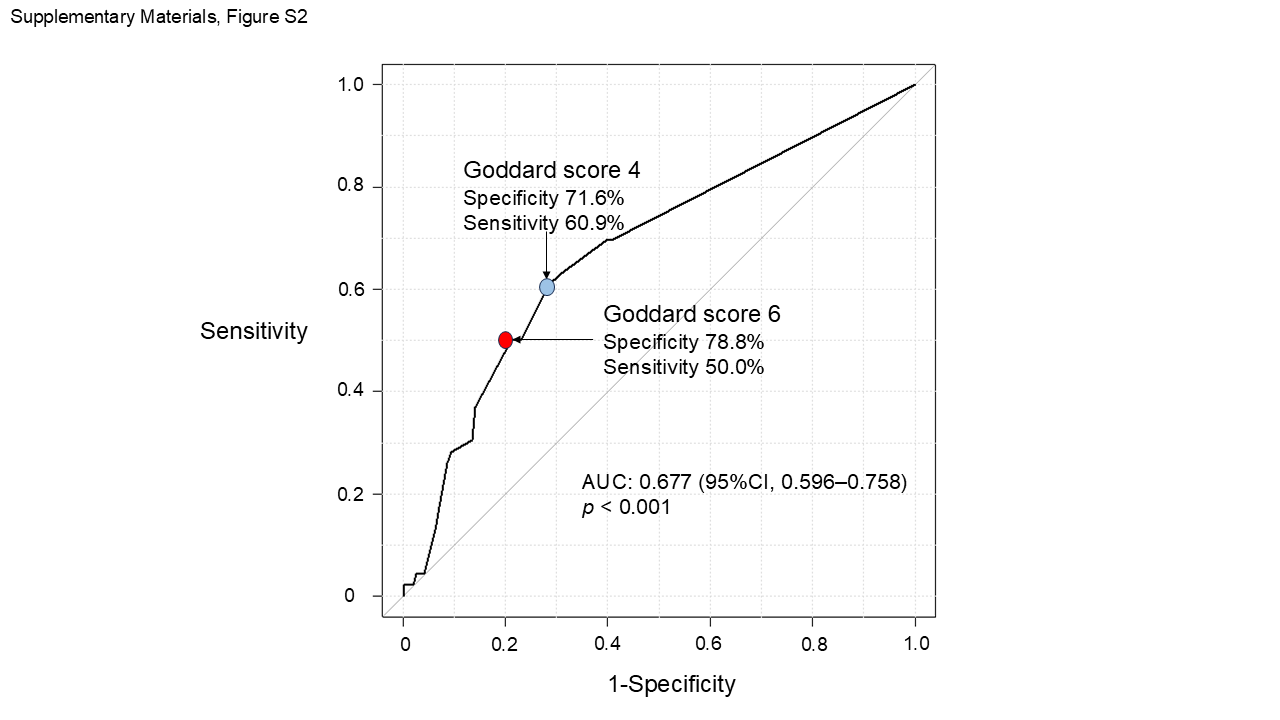

Supplement: ivag167_Supplementary_Data [file ivag167_supplementary_data.zip › Supplementary_Data/Figure S2.tif]

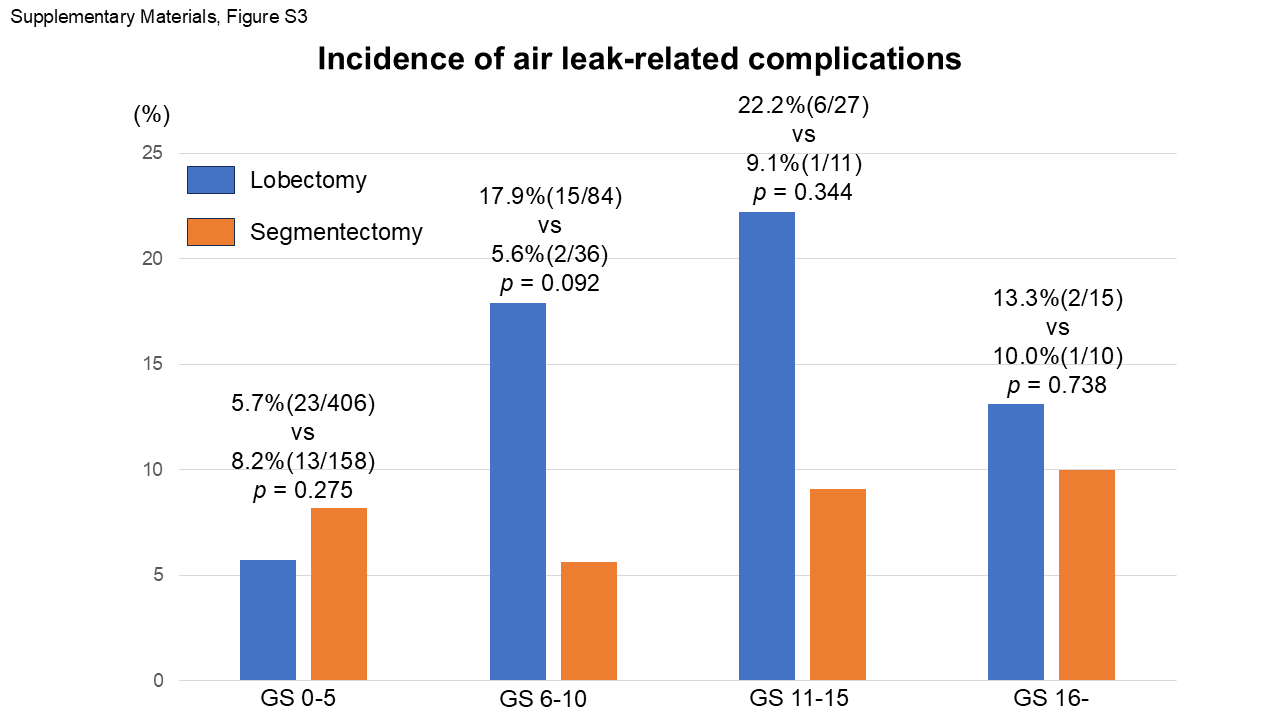

Supplement: ivag167_Supplementary_Data [file ivag167_supplementary_data.zip › Supplementary_Data/Figure S3.tif]
